# Supplementary material for: Alexithymia in Adult Autism Clinic Service-Users: Relationships with Sensory Processing Differences and Mental Health
Source: Healthcare (Basel). 2023 Dec 7;11(24):3114. doi: 10.3390/healthcare11243114 (PMC10742835; doi:10.3390/healthcare11243114)
Supplement: Supplementary file 1 [file healthcare-11-03114-s001.zip › healthcare-2712091-supplementary.pdf]

## Supplementary Materials

**Table S1.** *Diagnostic group characteristic and comparisons.*

| | Diagnosed | | | Not diagnosed | | | Group comparison | | | |
|---|---|---|---|---|---|---|---|---|---|---|
| **Demographic/clinical measure** | ***N*** | **Median (IQR)** | **Range** | ***N*** | **Median (IQR)** | **Range** | **$\chi^2$ (df, *N*)** | | ***p*** | ***φ*** |
| Sex: males (females) | 105 (48) | - | - | 19 (18) | - | - | 3.92 (1, 190) | | 0.05* | 0.14 |
| Presence of intellectual disability | 2 | - | - | 5 | - | - | Fisher's exact test | | 0.62 | - |
| | ***N*** | **Median (IQR)** | **Range** | ***N*** | **Median (IQR)** | **Range** | ***U*** | ***Z*** | ***p*** | ***r* effect size** |
| Age (years) | 152 | 32.0 (18.00) | 18-68 | 37 | 38.0 (21.00) | 18-65 | 2163.5 | -2.18 | 0.03* | 0.16 |
| AASP total | 96 | 176.5 (39.25) | 130-266 | 30 | 162.5 (33.75) | 129-241 | 1714.0 | 1.57 | 0.12 | 0.14 |
| Low registration (AASP) | 97 | 45.0 (15.50) | 23-71 | 31 | 40.0 (10.00) | 30-61 | 1061.0 | 2.47 | 0.02* | 0.21 |
| Sensation seeking (AASP) | 96 | 35.0 (11.00) | 19-55 | 30 | 39.5 (10.25) | 25-53 | 845.0 | -3.41 | <0.001*** | 0.30 |
| Sensory sensitivity (AASP) | 97 | 48.0 (16.50) | 21-75 | 31 | 43.0 (10.00) | 25-68 | 1213.5 | 1.62 | 0.12 | 0.14 |
| Sensation avoiding (AASP) | 96 | 51.0 (15.00) | 30-74 | 31 | 44.0 (15.00) | 21-69 | 1008.5 | 2.70 | 0.01** | 0.24 |
| Depression (HADS) | 122 | 10.0 (7.00) | 0-21 | 31 | 7.0 (8.00) | 0-19 | 1399.0 | 2.24 | 0.03* | 0.18 |
| Anxiety (HADS) | 123 | 12.0 (6.00) | 0-21 | 31 | 13.0 (8.00) | 4-21 | 1886.5 | 0.09 | 0.92 | 0.07 |
| AQ | 123 | 36.0 (11.00) | 17-50 | 29 | 34.0 (10.00) | 11-47 | 1468.5 | 1.48 | 0.14 | 0.12 |
| Alexithymia total (TAS-20) | 147 | 66.0 (16.00) | 30-94 | 35 | 65.0 (21.00) | 39-83 | 2301.5 | 0.97 | 0.33 | 0.07 |
| Difficulty describing feelings (TAS-20) | 153 | 20.0 (6.00) | 7-25 | 37 | 18.0 (8.50) | 9-24 | 2277.5 | 1.85 | 0.06 | 0.13 |
| Difficulty identifying feelings (TAS-20) | 150 | 24.0 (9.00) | 7-35 | 36 | 25.0 (7.75) | 10-35 | 2668.5 | -0.11 | 0.91 | 0.01 |
| Externally orientated thinking (TAS-20) | 149 | 23.0 (7.00) | 9-36 | 35 | 21.0 (7.00) | 10-34 | 2182.5 | 1.50 | 0.13 | 0.11 |

IQR, interquartile range; $\chi^2$ (df, *N*), chi-square test (degree of freedom, number of participants); $\varphi$, effect size $\sqrt{(\chi^2/N)}$; U, statistic from Mann-Whitney test; Z, standardised statistic from Mann-Whitney test; r, effect size $(Z/\sqrt{N})$; AQ, Autism Quotient; AASP, Adolescent/Adult Sensory Profile; HADS, Hospital Anxiety and Depression Scale; TAS-20, Toronto Alexithymia Scale. *Significant at $p \leq 0.05$; **Significant at $p \leq 0.01$; ***Bonferroni correction: significant at $p \leq 0.003$ (0.05/15)

**Table S2.** *Co-occurring conditions across the sample.*

| Co-occurring Conditions | Anxiety Conditions | Mood Conditions | ADHD | Obsessive-Compulsive and Related Conditions | Other NDCs | Intellectual Disability | Psychotic Conditions | Specific Learning Difficulties | Personality Conditions | Disordered Eating Conditions | Trauma and Stressor-Related Conditions |
|---|---|---|---|---|---|---|---|---|---|---|---|
| *N* | 132 | 91 | 36 | 33 | 7 | 7 | 6 | 5 | 4 | 4 | 4 |
| Percentage Frequency (%) | 69.5% | 47.9% | 18.9% | 17.4% | 3.7% | 3.7% | 3.2% | 2.6% | 2.1% | 2.1% | 2.1% |

ADHD, Attention Deficit Hyperactivity Disorder; NDC, Neurodevelopmental Conditions; *N*, number of participants. Conditions within each category are listed below:

**Anxiety Conditions:** Generalised Anxiety Disorder, Mixed Anxiety Disorder, Social Anxiety, Agoraphobia

**Mood Conditions:** Depressive Disorder, Dysthymia, Atypical Depressive Disorder, Post-Natal Depression, Major Affective Disorder, Bipolar Affective Disorder

**Obsessive-Compulsive and Related Conditions:** Obsessive-Compulsive Disorder, Hoarding Disorder, Trichotillomania

**Other NDCs:** Tourette's Syndrome, Tic Disorder, Social Communication Disorder, Dyspraxia

**Psychotic Conditions:** Schizophrenia, Delusional Disorder, Psychosis

**Specific Learning Difficulties:** Dyslexia, Dyscalculia

**Personality Conditions:** Schizoid Personality Disorder, Narcissistic Personality Disorder, Anti-Social Personality Disorder, Borderline Personality Disorder

**Disordered Eating Conditions:** Anorexia, Atypical Anorexia, Bulimia

**Trauma and Stressor-Related Conditions:** Post-Traumatic Stress Disorder

**Table S3**. *Example items for all measures*

| Factors | Example items |
|---|---|
| Difficulty describing feelings | "It is difficult for me to find the right words for my feelings" |
| Difficulty identifying feelings | "I have feelings that I can't quite identify" |
| Externally orientated thinking | "I prefer talking to people about their daily activities rather than their feelings" |

The Toronto Alexithymia Scale factors and example items [53]

| Factor | Example items |
|---|---|
| Low registration | "I don't smell things that other people say they smell" |
| Sensation seeking | "I enjoy how it feels to move about" |
| Sensory sensitivity | "I become frustrated when trying to find something in a crowded drawer or messy room" |
| Sensation avoiding | "I avoid or wear gloves during activities that will make my hands messy" |

The Adolescent/Adult Sensory Profile factors and example items [54]

| Factor | Example Items |
|---|---|
| Depression | "I feel as if I am slowed down" |
| Anxiety | "I feel tense or wound up" |

The Hospital Anxiety and Depression Scale factors and example items [57]

| Factor | Example items |
|---|---|
| Social skill | "I would rather go to a library than a party" |
| Attention switching | "I would prefer to do things the same way over and over again" |
| Attention to detail | "I usually notice car number plates or similar strings of information" |
| Communication | "Other people frequently tell me that what I've said is impolite, even though I think it is polite" |
| Imagination | "When I am reading a story, I find it difficult to work out the characters' intentions" |

The Autism Quotient factors and example items [58]

**Table S4.** *Bivariate Spearman's rank correlation between continuous measures in the whole sample, diagnosed group, and not diagnosed group.*

| All | | 1 | 2 | 3 | 4 | 5 | 6 | 7 | 8 | 9 | 10 | 11 | 12 |
|---|---|---|---|---|---|---|---|---|---|---|---|---|---|
| 1. | Age (years) | - | - | - | - | - | - | - | - | - | - | - | - |
| 2. | TAS-20 total | -0.14 | - | - | - | - | - | - | - | - | - | - | - |
| 3. | Difficulty describing feelings (TAS-20) | -0.16* | 0.75*** | - | - | - | - | - | - | - | - | - | - |
| 4. | Difficulty identifying feelings (TAS-20) | -0.01 | 0.86*** | 0.59*** | - | - | - | - | - | - | - | - | - |
| 5. | Externally orientated thinking (TAS-20) | -0.21** | 0.62*** | 0.25*** | 0.25*** | - | - | - | - | - | - | - | - |
| 6. | AQ | 0.11 | 0.36*** | 0.42*** | 0.32*** | 0.11 | - | - | - | - | - | - | - |
| 7. | AASP total | -0.02 | 0.40*** | 0.37*** | 0.48*** | 0.06 | 0.43*** | - | - | - | - | - | - |
| 8. | Low registration (AASP) | -0.15 | 0.45*** | 0.35*** | 0.45*** | 0.23* | 0.24** | 0.72*** | - | - | - | - | - |
| 9. | Sensation seeking (AASP) | 0.09 | -0.31*** | -0.34*** | -0.23* | -0.25** | -0.38*** | 0.02 | -0.10 | - | - | - | - |
| 10. | Sensory sensitivity (AASP) | 0.05 | 0.42*** | 0.42*** | 0.51*** | 0.02 | 0.48*** | 0.88*** | 0.58*** | -0.22* | - | - | - |
| 11. | Sensation avoiding (AASP) | -0.02 | 0.29*** | 0.32*** | 0.36*** | 0.02 | 0.54*** | 0.79*** | 0.37*** | -0.27** | 0.74*** | - | - |
| 12. | Depression (HADS) | 0.04 | 0.40*** | 0.27*** | 0.43*** | 0.15 | 0.18* | 0.34*** | 0.44*** | -0.28** | 0.39*** | 0.26** | - |
| 13. | Anxiety (HADS) | 0.21** | 0.36*** | 0.30*** | 0.52*** | -0.12 | 0.32*** | 0.55*** | 0.38*** | -0.14 | 0.57*** | 0.46*** | 0.54*** |
| Diagnosed | | | | | | | | | | | | | |
| 1. | Age (years) | - | - | - | - | - | - | - | - | - | - | - | - |
| 2. | TAS-20 total | -0.21** | - | - | - | - | - | - | - | - | - | - | - |
| 3. | Difficulty describing feelings (TAS-20) | -0.21** | 0.75*** | - | - | - | - | - | - | - | - | - | - |
| 4. | Difficulty identifying feelings (TAS-20) | -0.08 | 0.87*** | 0.59*** | - | - | - | - | - | - | - | - | - |

| | | | | | | | | | | | | |
|---|---|---|---|---|---|---|---|---|---|---|---|---|
| 5. Externally orientated thinking (TAS-20) | -0.26** | **0.60*** ** | 0.24** | 0.26** | - | - | - | - | - | - | - | - |
| 6. AQ | 0.19 | **0.34*** ** | **0.41*** ** | **0.31*** ** | 0.07 | - | - | - | - | - | - | - |
| 7. AASP total | 0.00 | **0.51*** ** | **0.44*** ** | **0.58*** ** | 0.09 | **0.46*** ** | - | - | - | - | - | - |
| 8. Low registration (AASP) | -0.11 | **0.50*** ** | **0.37*** ** | **0.50*** ** | 0.24* | 0.19 | **0.75*** ** | - | - | - | - | - |
| 9. Sensation seeking (AASP) | 0.05 | -0.27** | -0.32** | -0.12 | -0.23* | **-0.37*** ** | 0.10 | 0.10 | - | - | - | - |
| 10. Sensory sensitivity (AASP) | 0.04 | **0.52*** ** | **0.50*** ** | **0.60*** ** | 0.04 | **0.54*** ** | **0.87*** ** | **0.59*** ** | -0.20 | - | - | - |
| 11. Sensation avoiding (AASP) | 0.04 | **0.38*** ** | **0.38*** ** | **0.44*** ** | 0.01 | **0.61*** ** | **0.77*** ** | **0.33*** ** | -0.16 | **0.72*** ** | - | - |
| 12. Depression (HADS) | 0.00 | **0.35*** ** | 0.20* | **0.40*** ** | 0.10 | 0.12 | **0.41*** ** | **0.47*** ** | -0.17 | **0.43*** ** | 0.26* | - |
| 13. Anxiety (HADS) | 0.15 | **0.34*** ** | **0.30*** ** | **0.52*** ** | -0.18 | 0.29** | **0.57*** ** | **0.41*** ** | -0.12 | **0.58*** ** | **0.50*** ** | **0.49*** ** |
| **Not diagnosed** | | | | | | | | | | | | |
| 1. Age (years) | - | - | - | - | - | - | - | - | - | - | - | - |
| 2. TAS-20 total | 0.28 | - | - | - | - | - | - | - | - | - | - | - |
| 3. Difficulty describing feelings (TAS-20) | 0.21 | **0.75*** ** | - | - | - | - | - | - | - | - | - | - |
| 4. Difficulty identifying feelings (TAS-20) | 0.28 | **0.86*** ** | **0.63*** ** | - | - | - | - | - | - | - | - | - |
| 5. Externally orientated thinking (TAS-20) | 0.09 | **0.67*** ** | 0.26 | 0.31 | - | - | - | - | - | - | - | - |
| 6. AQ | 0.16 | 0.44* | 0.43* | 0.39* | 0.18 | - | - | - | - | - | - | - |
| 7. AASP total | -0.01 | 0.06 | 0.05 | 0.12 | -0.01 | 0.26 | - | - | - | - | - | - |
| 8. Low registration (AASP) | -0.14 | 0.36* | 0.31 | 0.37* | 0.19 | 0.40* | **0.64*** ** | - | - | - | - | - |
| 9. Sensation seeking (AASP) | 0.08 | -0.46* | -0.24 | **-0.56*** ** | -0.25 | -0.27 | -0.13 | -0.40* | - | - | - | - |
| 10. Sensory sensitivity (AASP) | 0.15 | 0.16 | 0.16 | 0.25 | -0.02 | 0.19 | **0.83*** ** | 0.46** | -0.23 | - | - | - |
| 11. Sensation avoiding (AASP) | -0.04 | 0.09 | 0.03 | 0.19 | -0.04 | 0.27 | **0.84*** ** | 0.45* | -0.43* | **0.75*** ** | - | - |

| | | | | | | | | | | | | |
|---|---|---|---|---|---|---|---|---|---|---|---|---|
| 12. Depression (HADS) | 0.29 | **0.59*** ** | 0.44* | **0.58*** ** | 0.23 | 0.40* | 0.15 | 0.33 | -0.37 | 0.32 | 0.22 | - |
| 13. Anxiety (HADS) | 0.40* | 0.44* | 0.34 | 0.45* | 0.10 | 0.49** | 0.49* | 0.36 | -0.33 | 0.55** | 0.47* | **0.78*** ** |

TAS-20, Toronto Alexithymia Scale; AQ, Autism Quotient; AASP, Adolescent/Adult Sensory Profile; HADS, Hospital Anxiety and Depression scale
*Significant at $p \le 0.05$; **Significant at $\le 0.01$; ***Bonferroni correction: significant at $p \le 0.001$ (0.05/468)

**Table S5.** *Descriptives and group comparisons of sex on key clinical measures in the whole sample.*

| | Males | | | Females | | | Group comparison | | | |
|---|---|---|---|---|---|---|---|---|---|---|
| **Clinical measure** | ***N*** | **Median (IQR)** | **Range** | ***N*** | **Median (IQR)** | **Range** | ***U*** | ***Z*** | ***p*** | ***r* effect size** |
| TAS-20 total | 121 | 65.0 (18.50) | 30-94 | 61 | 67.0 (14.00) | 33-93 | 3720.5 | 0.09 | 0.93 | 0.00 |
| Difficulty describing feelings (TAS-20) | 124 | 19.0 (5.75) | 7-25 | 66 | 20.0 (5.25) | 9-25 | 4410.0 | 0.88 | 0.38 | 0.06 |
| Difficulty identifying feelings (TAS-20) | 124 | 24.0 (9.00) | 7-25 | 62 | 25.0 (9.00) | 7-35 | 4243.0 | 1.15 | 0.25 | 0.08 |
| Externally orientated thinking (TAS-20) | 121 | 23.0 (6.50) | 9-34 | 63 | 22.0 (8.00) | 10-36 | 3360.5 | -1.32 | 0.19 | 0.09 |
| Sensory processing differences (AASP) | 85 | 165.0 (32.0) | 126-266 | 41 | 188.0 (44.0) | 131-241 | 2371.5 | 3.28 | 0.001*** | 0.29 |
| Depression (HADS) | 104 | 10.0 (7.00) | 1-21 | 49 | 8.0 (9.00) | 0-17 | 3474.0 | -1.17 | 0.24 | 0.09 |
| Anxiety (HADS) | 104 | 12.0 (6.75) | 0-21 | 50 | 13.0 (6.00) | 4-21 | 2807.5 | 0.80 | 0.42 | 0.06 |

IQR, interquartile range; $\chi^2$ (df, *N*), chi-square test (degree of freedom, number of participants); U, statistic from Mann-Whitney test; Z, standardised statistic from Mann-Whitney test; r, effect size ($Z/\sqrt{N}$); TAS-20, Toronto Alexithymia Scale; AASP, Adolescent/Adult Sensory Profile. HADS, Hospital Anxiety and Depression Scale.

*Significant at $p \leq 0.05$; **Significant at $p \leq 0.01$; ***Bonferroni correction: significant at $p \leq 0.004$ (0.05/14)

**Table S6.** *Descriptives and group comparisons of depression and anxiety severity for those with severe vs lower alexithymia in the whole sample.*

| | **Severe alexithymia (≥61)** | | | **Lower Alexithymia (<61)** | | | **Group comparisons** | | | |
|---|---|---|---|---|---|---|---|---|---|---|
| **Clinical measure** | ***N*** | **Median (IQR)** | **Range** | ***N*** | **Median (IQR)** | **Range** | ***U*** | ***Z*** | ***p*** | ***r* effect size** |
| Depression (HADS) | 98 | 10 (6) | 1-20 | 51 | 7 (8) | 0-21 | 3306.5 | 3.24 | 0.001*** | 0.27 |
| Anxiety (HADS) | 99 | 13 (6) | 3-21 | 51 | 11 (8) | 0-19 | 3223.0 | 2.78 | 0.005*** | 0.23 |

IQR, interquartile range; U, statistic from Mann-Whitney test; Z, standardised statistic from Mann-Whitney test; r, effect size (Z/√*N*); HADS, Hospital Anxiety and Depression Scale**.**

***Bonferroni correction: significant at p≤0.03 (0.05/2).

**Table S7.** *Depression and anxiety severity, regressed onto alexithymia severity and sensory processing differences, controlling for sex, diagnostic status, and anxiety/depression severity.*

| | | **Depression (HADS)**<br>β (95% CI) | **Anxiety (HADS)**<br>β (95% CI) |
|---|---|---|---|
| **Model 1 (*N*=105)** | Sex | -0.12 (-0.59 to 0.09) | 0.05 (-0.22 to 0.45) |
| | ASC diagnostic status | 0.18 (0.06 to 0.77)* | -0.18 (-0.78 to -0.08)* |
| | AASP | 0.04 (-0.15 to 023) | **0.32 (0.15 to 0.51)*** ** |
| | TAS-20 total | 0.19 (0.02 to 0.37)* | 0.11 (-0.06 to 0.29) |
| | Anxiety/Depression (HADS) | **0.50 (0.30 to 0.65)*** ** | **0.45 (0.29 to 0.64)*** ** |
| **Model fit ($R^2_{adj}$)** | | $F_{(5,99)}$ = 15.06, *p*<0.001 (40.3%) | $F_{(5,99)}$ = 19.11, *p*<0.001 (46.5%) |
| **Model 2 (*N*=107)** | Sex | -0.15 (-0.66 to 0.01) | 0.03 (-0.28 to 0.40) |
| | ASC diagnostic status | 0.16 (0.02 to 0.74)* | -0.19 (-0.81 to -0.10)* |
| | AASP | 0.05 (-0.16 to 0.24) | **0.34 (0.19 to 0.52)*** ** |
| | Difficulty describing feelings (TAS-20) | 0.18 (0.02 to 0.37)* | 0.09 (-0.08 to 0.28) |
| | Anxiety/Depression (HADS) | **0.51 (0.32 to 0.67)*** ** | **0.46 (0.31 to 0.65)*** ** |
| **Model fit ($R^2_{adj}$)** | | $F_{(5,101)}$ = 15.52, *p*<0.001 (40.7%) | $F_{(5,101)}$ = 19.44, *p*<0.001 (46.5%) |
| **Model 3 (*N*=107)** | Sex | -0.13 (-0.61 to 0.07) | 0.05 (-0.21 to 0.45) |
| | ASC diagnostic status | 0.21 (0.13 to 0.84)** | -0.14 (-0.69 to 0.01) |
| | AASP | 0.02 (-0.17 to 0.22) | 0.27 (0.10 to 0.45)** |
| | Difficulty identifying feelings (TAS-20) | 0.20 (0.01 to 0.38)** | 0.23 (0.06 to 0.41)** |
| | Anxiety/Depression (HADS) | **0.48 (0.28 to 0.65)*** ** | **0.41 (0.26 to 0.59)*** ** |
| **Model fit ($R^2_{adj}$)** | | $F_{(5,101)}$ = 15.36, *p*<0.001 (40.4%) | $F_{(5,101)}$ = 21.58, *p*<0.001 (49.3%) |

β (95% CI), regression coefficient and 95% confidence interval; F(df, df), analysis of variance statistic (regression degrees of freedom, residual degrees of freedom); ASC, Autism spectrum condition; HADS, Hospital Anxiety and Depression Scale; AQ, Autism Quotient; TAS-20, Toronto Alexithymia Scale

*Significant at p≤0.05; **Significant at p≤0.01; ***Bonferroni correction: significant at p≤0.002 (0.05/30).

**Table S8.** *Direct and indirect effects of difficulty describing feelings as a mediator of the relationship between sensory processing differences and depression severity, controlling for sex and diagnostic status.*

| Independent variable | Dependent variable | Effect | β | SE | LLCI | ULCI |
|---|---|---|---|---|---|---|
| AASP *N* = 107 | Depression (HADS) | Direct | 0.30* | 0.09 | 0.11 | 0.48 |
| | | Indirect via Difficulty describing feelings (TAS-20) | 0.10* | 0.04 | 0.03 | 0.19 |

β, regression coefficient; SE, Standard error; LLCI, Lower level confidence interval; ULCI, Upper level confidence interval; AASP, Adolescent/Adult Sensory Profile; HADS, Hospital Anxiety and Depression Scale; TAS-20, Toronto Alexithymia Scale. Indirect SE, LLCI and ULCI were bootstrapped.
*Significant at $p \leq 0.05$

**Table S9.** *Direct and indirect effects of difficulty identifying feelings as a mediator of the relationship between sensory processing differences and anxiety severity, controlling for sex and diagnostic status.*

| Independent variable | Dependent variable | Effect | β | SE | LLCI | ULCI |
|---|---|---|---|---|---|---|
| AASP *N* = 107 | Anxiety (HADS) | Direct | 0.36* | 0.10 | 0.16 | 0.55 |
| | | Indirect via Difficulty identifying feelings (TAS-20) | 0.22* | 0.06 | 0.11 | 0.36 |

β, regression coefficient; SE, Standard error; LLCI, Lower level confidence interval; ULCI, Upper level confidence interval; AASP, Adolescent/Adult Sensory Profile; HADS, Hospital Anxiety and Depression Scale; TAS-20, Toronto Alexithymia Scale. Indirect SE, LLCI and ULCI were bootstrapped. *Significant at $p \leq 0.05$
